# A population-based study of overweight and obesity in expectant parents: socio-demographic patterns and within-couple associations

**DOI:** 10.1186/1471-2458-13-923

**Published:** 2013-10-03

**Authors:** Kristina Edvardsson, Marie Lindkvist, Eva Eurenius, Ingrid Mogren, Rhonda Small, Anneli Ivarsson

**Affiliations:** 1 Department of Public Health and Clinical Medicine, Epidemiology and Global Health, Umeå University, SE 901 87 Umeå, Sweden; 2 Mother and Child Health Research, La Trobe University, Melbourne, Vic 3000, Australia; 3 Department of Statistics, Umeå University, SE 901 87 Umeå, Sweden; 4 Department of Clinical Sciences, Obstetrics and Gynecology, Umeå University, SE 901 87 Umeå, Sweden

**Keywords:** Body mass index, Childbirth, Child health, Cross-sectional studies, Fathers, Obesity, Overweight, Pregnant women, Socioeconomic factors

## Abstract

**Background:**

Overweight and obesity in pregnancy increase the risk of several adverse pregnancy outcomes. However, both mothers’ and fathers’ health play an important role for long-term health outcomes in offspring. While aspects of health and lifestyle of pregnant women have been reported, the health of expectant fathers and correlations of health variables within couples have received less attention. This study aimed to explore the prevalence and socio-demographic patterns of overweight and obesity in Swedish expectant parents, and to assess within-couple associations.

**Methods:**

This population-based, cross-sectional study investigated self-reported data from 4352 pregnant women and 3949 expectant fathers, comprising 3356 identified couples. Data were collected in antenatal care clinics between January 2008 and December 2011. Descriptive, correlation and logistic regression analyses were performed.

**Results:**

The self-reported prevalence of overweight (BMI 25.0-29.99) and obesity (BMI ≥30.0) was 29% among women (pre-pregnancy) and 53% among expectant fathers. In a majority of couples (62%), at least one partner was overweight or obese. The odds of being overweight or obese increased relative to partner’s overweight or obesity, and women’s odds of being obese were more than six times higher if their partners were also obese in comparison with women whose partners were of normal weight (OR 6.2, CI 4.2-9.3). A socio-demographic gradient was found in both genders in relation to education, occupation and area of residence, with higher odds of being obese further down the social ladder. The cumulative influence of these factors showed a substantial increase in the odds of obesity for the least compared to the most privileged (OR 6.5, CI 3.6-11.8).

**Conclusions:**

The prevalence of overweight and obesity in expectant parents was high, with a clear social gradient, and a minority of couples reported both partners with a healthy weight at the onset of pregnancy. Partner influence on health and health behaviours, and the role both mothers and fathers play in health outcomes of their offspring, underpin the need for a more holistic and gender inclusive approach to the delivery of pregnancy care and postnatal and child health services, with active measures employed to involve fathers.

## Background

It is well known that parent and child health are closely interlinked [1,2], and that parents’ health and health behaviours before conception and during pregnancy can influence the health of offspring [3-7]. Doyle and colleagues argue for the 'antenatal investment hypothesis’, that is, that the antenatal period is the stage when investment in early interventions gives the highest return [6]. They suggest that promoting parent health is an important 'investment’ strategy in efforts to promote child health because of the potential to prevent the influence of intergenerational risk factors with an associated impact on the socioeconomic gradient in health.

Overweight and obesity have become a major global health concern, and are now associated with more deaths worldwide than underweight [8]. The prevalence of overweight and obesity among pregnant women is increasing in many countries including Sweden [9,10]. Currently, 37% of women in Sweden are overweight or obese at the time of registration in antenatal care (ANC), which equates to a 48% increase over 17 years [10]. Overweight and obesity in pregnancy have been shown to increase the risk of adverse pregnancy outcomes such as gestational diabetes mellitus and hypertension, pre-eclampsia, thromboembolism, induced labour, caesarean delivery, preterm birth, postpartum haemorrhage, large-for-gestational-age (LGA), macrosomia, congenital abnormalities and also adversely affect initiation and duration of breastfeeding [11-19]. Furthermore, a Body Mass Index (BMI) ≥25 prior to pregnancy is the most important modifiable risk factor for stillbirths in high-income countries [20]. A link has also been shown to long-term adverse outcomes such as insulin resistance and cardiovascular risk factors in offspring [21-24], as well as autism, ADHD symptoms, developmental delay, and emotional difficulties [25-27]. Not only maternal, but also paternal overweight and obesity have been shown to increase the long-term risk for overweight and obesity in offspring, even though results are conflicting about which influence is stronger. Some studies suggest a stronger mother-offspring association [28,29], while others have shown stronger father-offspring associations [30], or similar strength of association for both parents [31-34].

Even though current evidence suggests that both parents are important for offspring health [1,30], the influence of expectant fathers, and correlations of health variables within expectant couples, have so far received little attention [35,36]. The aim of this study was to explore the prevalence and socio-demographic patterns of overweight and obesity among a population-based sample of expectant parents in Västerbotten, Sweden, and also to assess within-couple associations.

The research questions were: 1) What is the prevalence of overweight and obesity in expectant parents and couples, and what are the gender differences?, 2) Are there any within-couple associations of BMI?, and 3) What are the associations between socio-demographic characteristics, and overweight and obesity?

## Methods

### Design, population and data collection procedures

Data for this population-based, cross-sectional study were obtained via the Salut Programme, an ongoing child health promotion programme in the county of Västerbotten (260,000 inhabitants), Sweden. The programme was initiated by the County Council in 2005, and aims to support health promotion activities in ANC, child health care (CHC), dental services, social services, pre-schools and schools, and also to develop an epidemiological surveillance system. The Salut Programme has been further described elsewhere [37-40]. Data were collected between January 2008 and December 2011 through mailed questionnaires to pregnant women and their partners at enrolment in ANC. Women were identified as potential participants when they first contacted ANC to make a booking for their enrolment visit, where after the midwife sent the questionnaires to the pregnant woman and her partner (using the woman’s address). The questionnaires were returned on the day of enrolment (mean gestational age was 10 weeks calculated in relation to last menstrual period). The pregnant women’s questionnaires were used by midwives as a basis for health and lifestyle counselling, while partner questionnaires were used for epidemiological surveillance and research purposes only. Questionnaires included variables on socio-demographics, BMI, health and lifestyle (questions used in the study are presented in Additional files 1 and 2). No reminders were sent. Since 2006 use of the questionnaires has been rolled out across the county in a stepwise fashion in ANC clinics, reaching countywide implementation in mid-2010. The ANC services reach almost 100% of pregnant women in Sweden, which means that almost all pregnant women and their partners in the county have been invited to answer the questionnaire from mid-2010. Response rates were estimated to be 55% (n=4352) for pregnant women and 50% (n=3949) for their partners, based on the total number of pregnant women enrolled in ANC and taking the stepwise implementation of questionnaires into account. Female partner questionnaires (n=16) have been excluded for the purposes of this study, and the remaining male partners are hereafter called expectant fathers. The couples (n=3356) were linked through their personal identity number (participants were asked to provide their own and their partner’s personal identity number); however, because of incomplete data it was not possible to link all pregnant women and expectant fathers. Therefore, the total number of couples was lower than the number of pregnant women and expectant fathers.

### Variables

#### Dependent variable

BMI, defined as weight in kilograms divided by the square of height in metres (kg/m^2^) [8], was calculated using self-reported height and weight. Both partners were asked about their current weight and height (at enrolment), and the women were also asked to report their weight just prior to the pregnancy. The women’s pre-pregnancy weight was used when calculating BMI. We categorised BMI into underweight (<18.5), normal weight (18.5-24.99), overweight (25.0-29.99) and obesity (≥30.0) according to the definitions of The World Health Organization (WHO) [41].

#### Independent variables

*Socio-demographic data* were education, occupation, area of residence, country of birth, and the pregnant woman’s cohabitation status. We categorised *educational levels* into ≤9 years, 11–12 years (completed senior high school), >12 years, and a completed university degree; *occupational status* was categorised into 'employed’, 'studying’, 'parental leave/unpaid domestic work’, 'unemployed’, and 'sick leave/retirement’. We used the location of the ANC clinic as a proxy measure for *area of residence*. ANC clinics located in the two largest cities, Umeå and Skellefteå (including a radius of 15 kilometres), were defined as urban areas with a population density of 2300 and 1500 people per km^2^, respectively, [42], all other areas were defined as rural. *Country of birth* was dichotomised into 'Sweden’ or 'other’, and *women’s cohabitation* into 'with expectant father’ or 'other/single’.

### Statistical analyses

In addition to descriptive data, the Chi-square test for independence was used to explore differences between categorised variables (with Yates Continuity Correction for 2 by 2 tables), and the independent samples t-test was used to compare differences in mean scores between groups. Within-couple associations of BMI were investigated using Spearman rank order correlation. Logistic regression analyses were used to estimate crude odds ratios (OR) and confidence intervals (95% CI) for within-couple associations of overweight and obesity. The relationship between categorised BMI and categorical independent variables were investigated using Chi Square tests. Logistic regression analyses were then undertaken to estimate crude odds ratios and 95% CI for the association between socio-demographic factors and overweight/obesity. The analyses were repeated in a multivariate logistic regression analysis where age and socio-demographic factors were used as adjustment variables. Finally, logistic regression analyses were performed to estimate age-adjusted odds ratios and 95% CI for the association between eight different combinations of socio-demographic factors and overweight/obesity. In these analyses, all variables were dichotomised. Data for pregnant women and expectant fathers were combined to increase robustness of the analyses, particularly as preliminary analyses revealed similar patterns for women and men. Because a correlation of BMI within couples was found, an exchangeable correlation structure was assumed and the parameters were estimated with generalised estimating equations. In all regression analyses, the reference population was participants with BMI <25.0, thus, obese participants were excluded in regressions for overweight, and overweight participants were excluded in regressions for obesity. Interaction analyses for independent variables in relation to BMI revealed no interactions. Sample sizes decreased in multivariate analyses, as complete data for each participant was required for inclusion. Statistical significance was defined as p<0.05 or a 95% CI excluding 1.0. SPSS Statistics software (version 19) was used in all analyses.

**Table 1:** Socio-demographic characteristics of study participants

		**Pregnant women**		**Expectant fathers**	**p-value**
N		4352		3949	
Mean age (SD)		29.1 (5.1)		31.6 (6.0)	<0.001^2^
Range, years		15-46		14-64	
	**%**	***n***	**%**	***n***	
**Education**		4244		3852	
University degree	44.9	1904	29.7	1143	<0.001^3^
>12 years	13.6	576	14.5	558	
11-12 years (senior high school)	34.8	1477	49.8	1917	
≤9 years	6.8	287	6.1	234	
**Occupation**		4054		3784	
Employed	74.5	3020	86.0	3254	<0.001^3^
Studying	11.0	446	5.5	208	
Parental leave/unpaid domestic work	6.1	248	1.5	58	
Unemployed	7.1	287	5.8	221	
Sick leave/retirement	1.3	53	1.1	43	
**Area of residence**		3673		3119	
Urban	73.7	2708	74.1	2310	0.775^3^
Rural	26.3	965	25.9	809	
**Country of birth**		4305		4283^1^	
Sweden	90.9	3914	91.0	3896	0.970^3^
Other	9.1	391	9.0	387	
**Cohabitation with expectant father**	94.8	3982	-		

^1^Country of birth for men was reported via women’s questionnaires.

^2^Independent-samples t-test.

^3^Chi-square test for independence.

**Table 2:** Participant characteristics in comparison with selected variables from the general population in Västerbotten and Sweden, respectively

	**Pregnant women n=4352**	**Pregnant women**^**1 **^**(year 2011)**		**Women, 25–44 years**^**2**^		**Expectant fathers n=3949**	**Men, 25–44 years**^**2**^	
		**Västerbotten**	**Sweden**	**Västerbotten %**	**Sweden %**		**Västerbotten %**	**Sweden %**
Mean age (SD)	29.1 (5.1)^3^	30.0 (5.0)^4^	30.3 (5.3)^4^			31.6 (6.0)		
Range, years	15-46	16-46	13-53			14-64		
**Education**	**%**	**%**	**%**			**%**		
University degree	44.9	53.7^5^	51.3^5^	40.7	35.5	29.7	25.3	23.8
**Occupation**								
Employed	74.5	73.0	70.5	79.6	78.1	86.0	81.9	82.0
**Country of birth**								
Sweden	90.9	91.2	82.3			91.0		
Other	9.1	8.8	17.7	13.8	23.2	9.0	12.7	21.9

^1^The Swedish Maternal Health Care Register. The registers estimated coverage was 90% and 81% for Västerbotten and Sweden, respectively in 2011 (personal communication).

^2^Statistics Sweden [42].

^3^Age at enrolment in ANC.

^4^Age at childbirth.

^5^The figures from the Swedish Maternal Health Care Register include all women who have studied at a university, while the figures from Statistics Sweden and the Salut Programme only include those with at least three years of tertiary education or a university degree, respectively.

### Ethics

Study participation was based on informed consent, and the study complied with the Helsinki Declaration. Ethics approval was obtained from the Ethics Review Board of Umeå University, Sweden (Ref. 2010-63-31M).

## Results

### Characteristics of study participants

Socio-demographic characteristics of study participants are given in Table 1. Key variables are compared to the general population in Västerbotten and Sweden, respectively, in Table 2. A total of 4352 pregnant women and 3949 expectant fathers were included, and 3356 couples could be identified. The mean age of women was 29.1 years (SD 5.1) and expectant fathers were significantly older with a mean age of 31.6 years (SD 6.0). More pregnant women than expectant fathers reported a university degree (44.9% versus 29.7%) and were to a lesser extent in employment (74.5% versus 86.0%). Two thirds of participants resided in urban areas and a tenth were born outside Sweden. Most women were cohabiting with the expectant father (94.8%).

**Table 3:** Prevalence of overweight and obesity in women (pre-pregnancy) and in expectant fathers

		**Women, pre-pregnancy**		**Expectant fathers**	**p-value**
		**N=3728**		**N=3764**	
**BMI**^**1 **^**mean (SD)**		**23.7 (4.3)**		**25.7 (3.6)**	**<0.001**^**7**^
	**%**	**n**	**%**	**n**	
Underweight^2^	4.3	160	0.3	10	<0.001^8^
Normal weight^3^	66.6	2484	47.1	1780	
Overweight^4^	20.0	745	42.7	1615	
Obesity^5^	9.1	339	9.9	374	.
Overweight or obesity^6^	29.1	1084	52.6	1989	<0.001^8^

^1^Body Mass Index (kg/m^2^).

^2^BMI <18.5.

^3^BMI 18.5-24.99.

^4^BMI 25.0-29.99.

^5^BMI ≥30.0.

^6^BMI ≥25.0.

^7^ Independent-samples t-test.

^8^ Chi-square test for independence.

**Table 4:** **Prevalence of overweight and obesity in couples**^**1**^

	**In at least one partner in the couple**		**In both partners**	
	**%**	**n**	**%**	**n**
Overweight^2^	53.4	1490	9.8	273
Obesity^3^	15.5	431	2.0	55
Overweight or obesity^4^	62.3	1742	18.2	507

^1^2795 couples included in analysis. Analyses based on women’s pre-pregnancy BMI.

^2^BMI 25.0-29.99.

^3^BMI ≥30.0.

^4^BMI ≥25.0.

### Prevalence of overweight and obesity

As shown in Table 3, 29.1% of women (pre-pregnancy) and 52.6% of expectant fathers reported weights and heights which indicated that they were overweight or obese (BMI ≥25.0), with one in ten participants (9.1% of women 9.9% of expectant fathers) categorised as obese (BMI ≥30.0). The mean BMI was significantly higher in expectant fathers (25.7) than in women (23.7, pre-pregnancy) (Table 3).

**Table 5:** Odds of being overweight or obese in relation to partner weight

	**Pregnant women**^**1**^			
	**Odds ratio for overweight**		**Odds ratio for obesity**	
	**(95% CI)**^**2 **^**N=2552**		**(95% CI) N= 2233**	
	**n**	**OR**	**n**	**OR**
**Expectant fathers**				
Normal weight^3^	1273	1.0	1131	1.0
Overweight^4^	1091	1.6 (1.3-2.0)	928	1.8 (1.3-2.5)
Obesity^5^	188	2.8 (2.0-3.9)	174	6.2 (4.2-9.3)

^1^Analyses based on women’s pre-pregnancy BMI.

^2^Statistical significance was defined as a 95% CI excluding 1.0.

^3^BMI <25.0 (including underweight).

^4^BMI 25.0-29.99.

^5^BMI ≥30.0.

### Prevalence of overweight and obesity in couples

As shown in Table 4, overweight or obesity (BMI ≥25.0) was identified in 62.3% of couples, i.e. in at least one of the two partners, and in 18.2% of couples overweight or obesity was identified in both partners. The corresponding prevalence for obesity alone (BMI ≥30.0) was 15.5% in at least one of the two partners and 2.0% in both partners (Table 4).

**Table 6:** **Socio-demographic factors and the odds of overweight or obesity in pregnant women**^**1 **^**and expectant fathers**

	**Pregnant women**^**1**^							
	**Odds ratio for overweight**^**2**^				**Odds ratio for obesity**^**3**^			
	***(n)***	**Crude OR**	***(n)***	**Adjusted**^**4 **^**OR**	***(n)***	**Crude OR**	***(n)***	**Adjusted**^**4 **^**OR**
	***%***	***(95% CI)***^***5***^		***(95% CI)***	***%***	***(95% CI)***		***(95% CI)***
	**Overweight**				**Obesity**			
**Education**	*(3329)*		*(2479)*		*(2931)*		*(2175)*	
University degree	19.3	1.0		1.0	6.1	1.0		1.0
> 12 years	18.9	1.03 (0.8-1.3)		1.1 (0.8-1.4)	9.8	1.7 (1.2-2.4)		1.6 (1.1-2.5)
11-12 years (senior high school)	20.9	1.2 (1.01-1.5)		1.4 (1.1-1.7)	12.2	2.2 (1.7-2.9)		2.2 (1.6-3.1)
≤ 9 years	21.4	1.3 (0.9-1.8)		1.5 (0.93-2.4)	12.1	2.2 (1.4-3.5)		2.7 (1.5-4.9)
**Occupation**	*(3178)*		*(2479)*		*(2790)*		*(2175)*	
Employed	19.8	1.00		1.0	8.3	1.0		1.0
Studying	19.7	0.98 (0.7-1.3)		0.95 (0.7-1.3)	7.4	0.9 (0.6-1.3)		0.8 (0.5-1.4)
Parental leave/unpaid domestic work	21.0	1.1 (0.8-1.6)		0.9 (0.60-1.4)	9.3	1.2 (0.7-1.9)		0.9 (0.5-1.7)
Unemployed	20.3	1.1 (0.8-1.6)		1.2 (0.8-1.8)	14.5	1.9 (1.3-2.9)		1.7 (1.1-2.8)
Sick leave/retirement	11.6	0.7 (0.3-1.8)		0.5 (0.1-1.7)	25.6	3.6 (1.7-7.3)		3.5 (1.6-8.0)
**Area of residence**	*(2876)*		*(2479)*		*(2516)*		*(2175)*	
Urban	18.8	1.0		1.0	7.5	1.0		1.0
Rural	24.6	1.6 (1.3-1.9)		1.7 (1.4-2.1)	12.9	2.0 (1.6-2.6)		1.8 (1.3-2.4)
	**Expectant fathers**							
	**Odds ratio for overweight**				**Odds ratio for obesity**			
	***(n)***	**Crude OR**	***(n)***	**Adjusted**^**4 **^**OR**	***(n)***	**Crude OR**	***(n)***	**Adjusted**^**4 **^**OR**
	***%***	***(95% CI)***		***(95% CI)***	***%***	***(95% CI)***		***(95% CI)***
	**Overweight**				**Obesity**			
**Education**	*(3331)*		*(2453)*		*(2108)*		*(1549)*	
University degree	40.0	1.0		1.0	5.9	1.0		1.0
> 12 years	44.6	1.6 (1.1-1.7)		1.5 (1.1-1.9)	10.6	2.1 (1.5-3.2)		1.6 (0.97-2.5)
11-12 years (senior high school)	44.4	1.4 (1.2-1.6)		1.6 (1.3-1.9)	11.5	2.4 (1.8-3.2)		1.8 (1.3-2.6)
≤ 9 years	41.4	1.3 (0.9-1.7)		1.4 (0.98-2.1)	13.8	2.8 (1.7-4.6)		2.2 (1.2-4.0)
**Occupation**	*(3268)*		*(2453)*		*(2073)*		*(1549)*	
Employed	43.0	1.0		1.0	9.5	1.0		1.0
Studying	42.8	0.9 (0.7-1.3)		0.98 (0.7-1.4)	6.7	0.7 (0.4-1.2)		0.6 (0.3-1.3)
Parental leave/unpaid domestic work	38.2	0.8 (0.4-1.3)		1.03 (0.6-1.9)	5.5	0.5 (0.2-1.6)		0.8 (0.2-2.7)
Unemployed	37.7	0.9 (0.7-1.2)		0.95 (0.7-1.4)	15.6	1.7 (1.1-2.6)		1.9 (1.1-3.1)
Sick leave/retirement	61.1	4.9 (1.8-12.9)		3.0 (1.1-8.3)	25.0	9.0 (3.0-27.1)		4.0 (1.2-14.0)
**Area of residence**	*(2705)*		*(2453)*		*(1703)*		*(1549)*	
Urban	43.1	1.0		1.00	7.9	1.0		1.0
Rural	42.8	1.1 (0.95-1.3)		1.04 (0.9-1.3)	14.2	2.0 (1.6-2.7)		1.9 (1.4-2.5)

^1^Pre-pregnancy BMI.

^2^BMI 25.0-29.99.

^3^BMI ≥30.0.

^4^Adjusted OR includes age, education, occupation and area of residence.

^5^Statistical significance was defined as a 95% CI excluding 1.0.

### Within-couple associations

We found a positive partner correlation for BMI (Spearman rho=0.21, p=<0.001). Furthermore, as shown in Table 5, the odds of reporting overweight or obesity increased relative to partner’s overweight or obesity. For example, there was a 6.2-fold greater odds for pregnant women who had an obese partner to report obesity, in comparison with women whose partners reported normal weight (OR 6.2, CI 4.2-9.3) (Table 5).

**Table 7:** **Odds of overweight or obesity in expectant parents**^**1 **^**for different combinations of socio-demographic risk factors**

**Nr**	**Combination of factors**							
	**Education**	**Occupation**	**Area of residence**	**No of risk factors**	**N=4933**	**OR**^**2 **^**Overweight**^**3**^	**N=3725**	**OR**^**2 **^**Obesity**^**4**^
						**(95% CI)**^**5**^		**(95% CI)**
					**n**		**n**	
**1**	University degree	Employed/studying/parental leave/unpaid domestic work	Urban	0	1637	1.0	1284	1.0
**2**	University degree	Employed/studying/parental leave/unpaid domestic work	Rural	1	299	1.4 (1.1-1.8)	225	1.7 (1.1-2.7)
**3**	University degree	Unemployed/sick leave/retirement	Urban	1	61	1.1 (0.6-1.9)	53	2.6 (1.3-5.2)
**4**	University degree	Unemployed/sick leave/retirement	Rural	2	15	0.9 (0.3-2.8)	13	1.2 (0.2-8.3)
**5**	Below university degree	Employed/studying/parental leave/unpaid domestic work	Urban	1	1851	1.8 (1.5-2.1)	1328	1.9 (1.5-2.5)
**6**	Below university degree	Employed/studying/parental leave/unpaid domestic work	Rural	2	832	2.2 (1.8-2.7)	620	3.9 (2.9-5.2)
**7**	Below university degree	Unemployed/sick leave/retirement	Urban	2	169	2.2 (1.6-3.1)	132	4.3 (2.7-6.9)
**8**	Below university degree	Unemployed/sick leave/retirement	Rural	3	69	1.6 (0.9-2.7)	70	6.5 (3.6-11.8)

^1^Analyses based on women’s pre-pregnancy BMI.

^2^Age adjusted OR.

^3^BMI 25.0-29.99.

^4^BMI ≥30.0.

^5^Statistical significance was defined as a 95% CI excluding 1.0.

### Influence of socio-demographic factors on overweight and obesity

Significant associations were found between BMI and education, occupation, and area of residence, respectively, for both genders, but not between BMI and country of birth or pregnant women’s cohabitation status, respectively. We found a gradient in the odds of obesity for both women and expectant fathers in relation to educational level, occupational status and area of residence (Table 6). The odds for pregnant women with short education (≤ 9 years) to be identified with obesity was 2.7 times higher (CI 1.5-4.9) compared to those with university education, after adjusting for age, occupation and area of residence. This gradient related to educational level was less pronounced for expectant fathers. The association between occupation and obesity showed that there was a 4-fold greater odds for expectant fathers on sick leave or early retired to report obesity than those in employment (adjusted OR 4.0, CI 1.2-14.0), and the equivalent adjusted OR for pregnant women was 3.5 (1.6-8.0). Living in rural areas doubled the odds of obesity in both genders, while in relation to overweight (BMI 25.0-29.99), this factor only showed an influence in pregnant women (adjusted OR 1.7, CI 1.4-2.1). See Table 6 for further details.

As shown in Table 7, when analysing the cumulative influence of identified risk factors in pregnant women and expectant fathers, we found that the odds of reporting obesity were almost seven times higher among the least privileged compared to those in the most privileged group (OR 6.5, CI 3.6-11.8). There was a clear and stepwise socio-demographic gradient in the odds of reporting obesity among those with education below university degree (groups no. 5–8). Those with a university degree but living in rural areas (group no. 2), as well as those unemployed, on sick leave or retired (group no. 3), also had higher odds of being identified as obese. The socio-demographic pattern was not as strong in the overweight category, although those with education below a university degree, and those with a university degree living in rural areas (groups no. 2 and 5–7), were found to be significantly more disadvantaged.

## Discussion

Four main findings emerged from this study. First, overweight and obesity were prevalent in one third of women just prior to pregnancy and in more than half of expectant fathers. Second, in a majority of couples, at least one partner was overweight or obese. Third, BMI was correlated within couples, and the odds of being overweight or obese increased relative to partner’s overweight or obesity, with women having more than six times the odds of reporting obesity if their partners were obese, in comparison with women whose partners reported normal weight. Lastly, a clear stepwise socio-demographic pattern was found in relation to obesity, with higher odds of being obese further down the social ladder. This pattern was not as strong in relation to overweight.

The combined prevalence of overweight and obesity in this study is similar to that previously reported from selected areas in Västerbotten [37], while the prevalence of obesity was lower for both women and expectant fathers. However, university education was more common in our study sample, which possibly explains these differences. Although the overweight prevalence was considerably higher among expectant fathers, almost as many women as expectant fathers reported obesity, based on their pre-pregnancy weight. This gender patterning is consistent with that reported in Sweden and internationally [43,44].

Our finding that at least one of the two partners reported overweight or obesity in a majority of couples has important long-term implications, as the prevalence of offspring overweight and obesity has been shown to be low in families with two normal-weight parents, while the risk gradually increases depending on whether one or both parents are overweight or obese as well as with increasing weight of the parents [7,28,30,45,46]. A recent Finnish study found an extremely high risk for overweight at age 16 years for children with both parents overweight or obese pre-pregnancy and at follow up, with a fifteen-fold increase for girls, and a six-fold increase for boys [7]. If these Finnish findings are generalisable, one fifth of children born in Västerbotten would be experiencing these same high risks. This evidence also points to the need to start obesity prevention as early as possible, i.e. already during pregnancy [47,48], or most ideally even before conception, and to include partners in delivery of health services at this time.

The correlation of BMI within couples found in this study was similar to that reported in other populations [49,50]. The correlation has also been shown previously to be strongest during the first years of cohabitation [50]. A previous study of expectant parents did not find within-couples correlation of BMI [51], though this might have been due to the small study sample. Partner resemblance in BMI may be explained by the tendency of people to select partners with similar educational level, income, health and health behaviours [52], shared environments [53], and by partner influence on an individual’s health behaviour change [54]. In a recent study, Berge and colleagues found the health promoting behaviours and attitudes of 'significant others’, e.g. boyfriend/girlfriend or partner, to be positively associated with health behaviours in young adult women and men, and that this could reduce the likelihood of overweight and obesity in young women [55]. Thus, involving the partner in health behaviour change could be one important strategy in reducing the risk of adverse pregnancy outcomes related to maternal overweight and obesity. Several studies have shown that even though expectant fathers want involvement in ANC [56], they often feel left out [39,57,58]. Thus, barriers for reaching expectant fathers or partners may be less related to lack of interest, than to other factors such as the organisation of services. However, further research is needed.

The clear socio-demographic gradients found in relation to obesity have also been reported in other studies [43,59-61], where people with shorter education were more likely to be obese than those with longer education, and where obesity was more common in rural than in urban areas. This has intergenerational implications as evidence suggests that such health inequalities will be mirrored in offspring health. We did not find that pregnant women or expectant fathers born outside Sweden had higher odds of being overweight or obese, which contradicts previous findings where immigrant women and men have shown an increased risk for obesity [62-64]. Further evidence is needed in relation to immigration patterns and overweight/obesity.

Expectant parents’ own awareness of maternal and offspring risks associated with parent overweight and obesity seems to be an understudied area [65]. In a previous study, all participating women were unaware of maternal and offspring risks, and informed discussions with health care professionals were lacking [66]. Other recent studies have also indicated low levels of awareness among women, especially in relation to adverse neonatal and offspring outcomes [65,67]. This seem consistent with findings from one of our previous studies, in which first time mothers and fathers frequently discussed pregnancy risks associated with smoking, alcohol, and toxins and germs in food, but very rarely mentioned maternal or offspring risks associated with overweight and obesity [39]. Knowledge about risks has been shown to be lower among women with shorter education [67], precisely the groups where overweight and obesity are more common [43,59-61]. It is also common that women do not recognise themselves as being overweight or obese [68], and it seems reasonable to interpret that this may also contribute to an underestimation of associated risks. Theories on health behaviour change suggest awareness of negative outcomes to be a crucial factor in individuals’ decisions to adapt healthier behaviours [69]. Thus, raising awareness among both women and men is likely to be one important and necessary component in efforts to reduce weight-related adverse maternal and offspring outcomes. However, raising issues about weight can be challenging for health care professionals [70,71], and although providers may perceive their counselling to be adequate, few women report discussing risks or receiving appropriate counselling about weight gain in pregnancy [65,72-74].

### Methodological considerations

The strengths of this study are the large population-based sample, which enhances the external validity of the study findings, the inclusion of expectant fathers, and also that couples could be identified. The pregnant women had a similar mean age and mean BMI at enrolment as the general pregnancy population in Västerbotten and Sweden [10,75]. The proportion of overweight expectant fathers was also consistent with previous reports for Swedish men aged 30–44 years [43]. However, study participants had on average a higher level of education than the general population aged 25–44 in Västerbotten and Sweden, and were less likely to be born outside Sweden. Previous studies have shown that low socio-economic groups are over-represented among non-respondents in population-based studies [76,77], and these groups are also more likely to have poorer lifestyle and health than respondents [76]. This phenomenon is also likely in this study given the difference in educational status between the study population and the general population in Västerbotten and Sweden. Information on BMI was missing for 14.3% of pregnant women and 4.7% of expectant fathers, resulting in exclusion from analyses. We found that the excluded women were younger, with a significantly lower proportion having a university degree, and they were significantly more often in employment compared to the whole sample of women. Correspondingly, the excluded expectant fathers contained a significantly lower proportion with a university degree and were significantly less likely to be in employment.

An overestimation of height and an underestimation of weight has been common in self-report studies among both women and men [78,79]. This would mean that the true prevalence of overweight and obesity is most probably even higher than reported in this study, especially considering that the educational status was higher in our study population, and that missing data on BMI were more common among those with lower educational levels. Thus, our findings are likely conservative prevalence estimates. In addition, the increasing problem of low response rates in epidemiologic research, threatens internal validity due to the risk of self-selection bias [80]. Our response rates, 55% and 50% for pregnant women and expectant fathers, are comparable to other studies in the field. Nevertheless, these results need to be interpreted with caution due to the self-reported nature of the data and the risk of selection bias.

## Conclusions

The prevalence of overweight and obesity in expectant parents was high, with a clear social gradient, and a minority of couples reported both partners with a healthy weight at the onset of pregnancy. Partner influence on health and health behaviours, and the role both mothers and fathers play in health outcomes of their offspring, underpin the need for a more holistic and gender inclusive approach to the delivery of pregnancy care and postnatal and child health services, with active measures employed to involve fathers.

## Competing interests

The authors declare that they have no competing interests.

## Authors’ contributions

KE, ML, EE, IM and AI designed the study. KE and ML conducted the analyses and KE drafted the manuscript. RS read and revised the manuscript with specific attention to language and clarity of presentation. All authors contributed in the writing process and approved the final manuscript.

## Pre-publication history

The pre-publication history for this paper can be accessed here:

http://www.biomedcentral.com/1471-2458/13/923/prepub

## Supplementary Material

Additional file 1 Excerpts from the pregnant woman’s questionnaire. Click here for file

Additional file 2 Excerpts from the expectant father’s questionnaire. Click here for file
