# Supplementary material for: A population-based study of overweight and obesity in expectant parents: socio-demographic patterns and within-couple associations
Source: BMC Public Health. 2013 Oct 3;13:923. doi: 10.1186/1471-2458-13-923 (PMC3854510; doi:10.1186/1471-2458-13-923)
Supplement: Additional file 1 — Excerpts from the pregnant woman’s questionnaire. [file 1471-2458-13-923-S1.pdf]

Additional file 1.

Today's date: .... / .... / ....
Year Month Day

Health centre: ....

Midwife: ....

Name:...

Personal identification number: [| | | | |] [| | |]

Street address:...

Postal code:... Locality: ....

Telephone: *home:*......................... *work:*................................. *mobile:*...........................

X1. What is your present type of occupation?

- ☐ Employed
- ☐ Self-employed
- ☐ Jobseeker for <u>more than</u> 6 months
- ☐ Jobseeker for <u>less than</u> 6 months
- ☐ Student, apprentice
- ☐ Doing household work at home (no personal income)
- ☐ On parental or other leave
- ☐ On sickness, old age or disability benefit

X3. What is the highest level of education you have completed?

- ☐ Less than 9 years of school
- ☐ Completed compulsory school, or the equivalent of 9 years of school
- ☐ Completed secondary school, or the equivalent of 12 years of school
- ☐ At least 1 year of school beyond secondary school
- ☐ A university degree

X4. In which country were you born?

- ☐ Sweden
- ☐ Another country, namely:..........................

X5. In which country was your partner born?

- ☐ Sweden
- ☐ Another country, namely:..........................

X6. With whom do you live?

☐ The father to be ☐ Another partner ☐ Single ☐ Other

How much do you weigh at present? appr. .... kg
How much did you weigh right <u>before</u> you became pregnant? appr. ....kg

How tall are you? appr. .... cm

Next of kin: Name:...

Personal identification number: [| | | | |] [| | |]

Address same as above ☐ Yes ☐ No
